# A refined genome phage display methodology delineates the human antibody response in patients with Chagas disease

**DOI:** 10.1016/j.isci.2021.102540

**Published:** 2021-05-15

**Authors:** André Azevedo Reis Teixeira, Luis Rodriguez Carnero, Andréia Kuramoto, Fenny Hui Fen Tang, Carlos Hernique Gomes, Natalia Bueno Pereira, Léa Campos de Oliveira, Regina Garrini, Jhonatas Sirino Monteiro, João Carlos Setubal, Ester Cerdeira Sabino, Renata Pasqualini, Walter Colli, Wadih Arap, Maria Júlia Manso Alves, Edécio Cunha-Neto, Ricardo José Giordano

**Affiliations:** 1 Department of Biochemistry, Institute of Chemistry, University of São Paulo, São Paulo, SP, 05508-000, Brazil; 2 Heart Institute (InCor), University of São Paulo School of Medicine, São Paulo, SP, 05403-000, Brazil; 3 Institute of Tropical Medicine, University of São Paulo School of Medicine, São Paulo, SP, 05403-000, Brazil; 4 Rutgers Cancer Institute of New Jersey, Newark, NJ 07103, USA; 5 Division of Cancer Biology, Department of Radiation Oncology, Rutgers New Jersey Medical School, Newark, NJ 07103, USA; 6 Division of Hematology/Oncology, Department of Medicine, Rutgers New Jersey Medical School, Newark, NJ 07103, USA; 7 Division of Clinical Immunology and Allergy, University of São Paulo School of Medicine, São Paulo, SP 01246-903, Brazil; 8 Institute for Investigation in Immunology (iii), INCT, São Paulo, SP, Brazil

**Keywords:** Parasitology, Sequence Analysis, Systems Biology

## Abstract

Large-scale mapping of antigens and epitopes is pivotal for developing immunotherapies but challenging, especially for eukaryotic pathogens, owing to their large genomes. Here, we developed an integrated platform for genome phage display (gPhage) to show that unbiased libraries of the eukaryotic parasite *Trypanosoma cruzi* enable the identification of thousands of antigens recognized by serum samples from patients with Chagas disease. Because most of these antigens are hypothetical proteins, gPhage provides evidence of their expression during infection. We built and validated a comprehensive map of Chagas disease antibody response to show how linear and putative conformation epitopes, many rich in repetitive elements, allow the parasite to evade a buildup of neutralizing antibodies directed against protein domains that mediate infection pathogenesis. Thus, the gPhage platform is a reproducible and effective tool for rapid simultaneous identification of epitopes and antigens, not only in Chagas disease but perhaps also in globally emerging/reemerging acute pathogens.

## Highlights

• Genomic shotgun phage display (gPhage) of eukaryotes is feasible and promising.
• gPhage allows rapid antigen ID and epitope mapping, including 3D structures.
• Conformation epitopes can be identified and validated by using the gPhage platform.
• Most Chagas disease antigens are hypothetical proteins rich in repetitive elements.

## Graphical abstract

**Figure:**
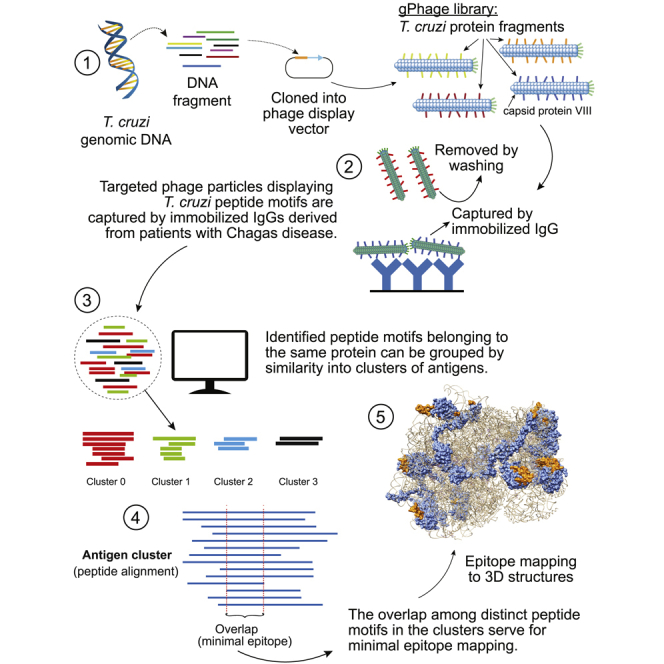

## Introduction

The repertoire of antigens and epitopes specifically recognized by antibodies represents the immunological memory for specific pathogens and diseases. Thus, antigen identification and epitope mapping are both crucial steps toward the development of vaccines, diagnostic tests, and therapeutic options for infectious and parasitic diseases. Chagas disease, leishmaniasis, and malaria, along with emerging multidrug resistance species of yeast (e.g., *Candida auris*) (Satoh et al., 2009; Caceres et al., 2019), are examples of diseases caused by eukaryotic infectious agents for which we do not have either vaccines or optimal therapeutics and, therefore, still challenge contemporary medicine and health systems worldwide. Along with them, the 21st century has seen a succession of viral pandemics, including the Zika and chikungunya virus, and respiratory viruses such as influenza H1N1, and coronavirus-induced severe respiratory syndromes, including severe acute respiratory syndrome and coronavirus disease 2019 (COVID-19). With the arrival of these global pandemics comes an unmet need for the rapid identification of antigens relevant for diagnosis and vaccine development.

Large-scale mapping of antibody repertoires—especially against pathogens with large genomes—can be particularly challenging. Such endeavors have long relied on a wide range of techniques, from assays such as enzyme-linked immunosorbent assay (ELISA), to cDNA libraries and microarray platforms (Sahin et al., 1995; Sutandy et al., 2013; El-Manzalawy et al., 2017). All these technologies are rather laborious and require expensive and specialized reagents: custom arrays require cloning and expression of thousands of proteins or designing and custom-ordering proprietary arrays of peptides (Sutandy et al., 2013). First, genes that have not yet been identified or characterized are not necessarily included in such assays, a problem that is prevalent for protozoan parasites, given that most of their genes are still classified as either hypothetical or predicted (El-Sayed et al., 2005; Howick et al., 2019). Moreover, many full-length proteins are not necessarily expressed well, which also may not fold properly, potentially masking antigenic epitopes. Synthetic peptides are limited to oligomers of specific lengths, potentially failing to sample conformational epitopes that require peptides with lengths outside the parameters of the array. Finally, once an antigen has been identified, it is necessary to carry out epitope mapping, which often involves the use of expensive peptide tiling and/or arrays. Here, we show that phage display integrated with computational approaches may potentially address many of these methodological shortcomings, allowing for the development of a less cumbersome, more cost-effective, and unbiased platform for simultaneous antigen identification and epitope mapping.

**Figure 1:**
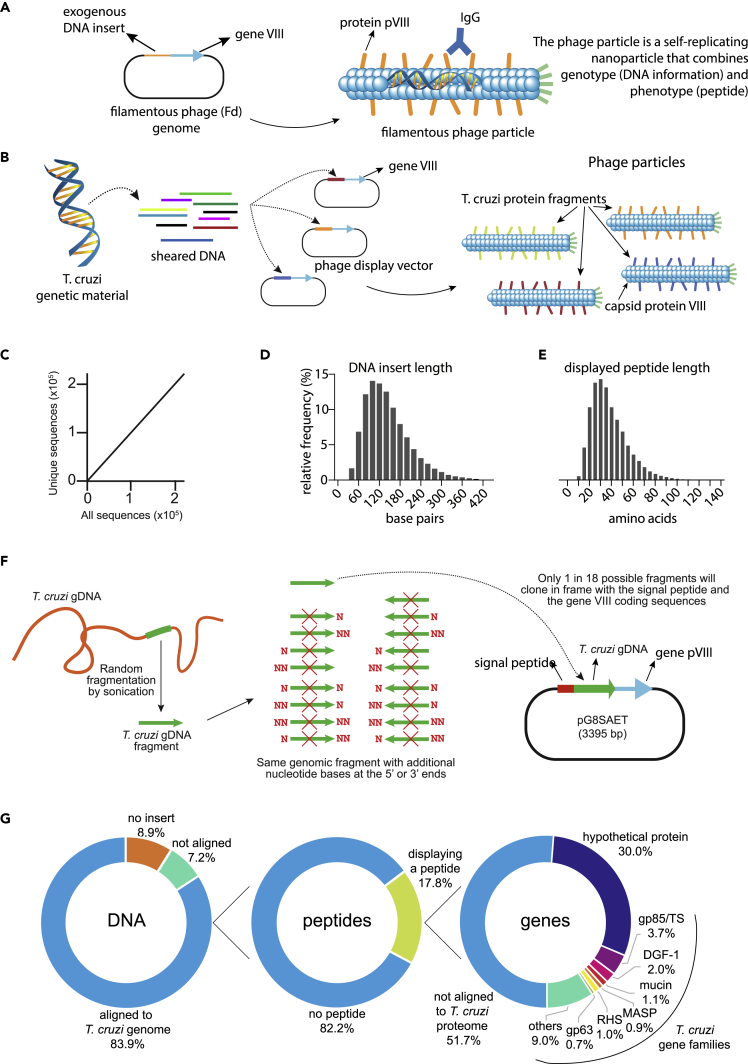
Building a gPhage library (A) Phage is a self-replicating nanoparticle that combines genotype (DNA information) and phenotype (peptide), including the epitope of an IgG. (B) *T*. *cruzi* genome is fragmented and cloned into the gPhage vector to produce a collection of phage particles, each displaying a *T*. *cruzi*-derived peptide. (C–E) gPhage library quality control. (C) The plot shows the number of distinct reads observed in the gPhage library as a function of the total number of sequences evaluated after filtering. The plots indicate length of DNA inserts (D) and corresponding peptides (E) in the gPhage library. (F) Cartoon illustrating the odds of a random gDNA inserts yield a phage particle displaying a *T*. *cruzi*-derived peptide. (G) Pie chart with gPhage library insert distribution: percentage of transformants containing a *T*. *cruzi*-derived insert (**left circle**); inserts that resulted in a peptide displayed on the surface of the bacteriophage particle (**central circle**), and finally, percentage of peptides that either match or do not match a *T*. *cruzi* protein in databanks.

Phage display has initially been developed and used to map antibody epitopes (Smith, 1985, 2019), identify antigens involved in diseases such as cancer (Mintz et al., 2015), autoimmune illnesses (Zhang and Reichlin, 2005), and parasitic infections (Ellis et al., 2012), including Chagas disease (Alvarez et al., 2001; Pitcovsky et al., 2001; Kamath et al., 2020). It has also been used for many other translational applications (Giordano et al., 2001; Arap et al., 2002; Michaloski et al., 2016; Tang et al., 2019), attesting to the great versatility of this combinatorial methodology. In its essence, phage display involves inserting a fragment of DNA in-frame with a bacteriophage surface coat protein gene, such that the corresponding peptide (or antibody) encoded by the exogenous DNA fragment is displayed on the bacteriophage surface (Figure 1A). If the peptide is a fragment of an antigen recognized by a given antibody, the bacteriophage particle can be captured and isolated from the pool of phage particles. The challenge with standard phage display is that random peptide libraries (produced with synthetic degenerate oligonucleotides) often lead to mimotopes, peptides that mimic but do not necessarily share sequence identity or similarity with the corresponding antigen. Despite these limitations, it has been instrumental in identifying relevant antigens and epitopes for several diseases, including Chagas disease (Pitcovsky et al., 2001; Mintz et al., 2015; Kamath et al., 2020). We have reasoned that this outcome could perhaps be circumvented with libraries produced from genetic material (cDNA or genomic DNA) isolated from an organism. These natural phage display libraries yield peptides encoded by the genome of the selected organism. Thus, when using genomic DNA, the result might perhaps be an unbiased phage library that displays peptides derived from all possible reading frames of a given organism genome. To date however, genome shotgun phage display libraries have been limited to viruses and prokaryotic organisms, given their comparatively smaller and far less complex, intronless genomes (Zhang et al., 1999; Jacobsson et al., 2003; Ching et al., 2012; Xu et al., 2015).

Here, we show that despite their larger and more complex genomes, genomic shotgun phage display libraries of eukaryotic organisms are possible to construct, express, and analyze and are capable of identifying antigens and epitopes. By combining phage display with large-scale sequencing and antigen discovery bioinformatic analytical tools, including protein structural data, we have developed genome phage display, henceforth termed gPhage, as an integrated platform for high-throughput antigen discovery and epitope mapping. Chagas disease is a tropical neglected illness caused by the unicellular protozoan parasite *Trypanosoma cruzi* and afflicts millions of patients worldwide (Pérez-Molina and Molina, 2018). As an experimental proof-of-concept for the gPhage platform introduced here, we have built a comprehensive map of the antibody response of patients suffering from this tropical neglected disease, in the acute and chronic stages of the disease.

Several methodologies have been successfully used to identify antigens and map relevant epitopes associated with Chagas disease. Early studies with cDNA expression libraries led to the identification of immunodominant antigens such as SAPA, TSA1, B13, mucins, and mucin-associated surface proteins (MASP), many of which are still used today for diagnostic purposes (Ibañez et al., 1988; Peterson et al., 1986; Duranti et al., 1999; Pollevick et al., 2000; Pitcovsky et al., 2001; De Pablos et al., 2016). Phage display technology has also been successfully used to map conformational epitopes in the gp85/trans-sialidase family of proteins (Pitcovsky et al., 2001), the SAPA antigen (Alvarez et al., 2001), and to identify mimotopes with diagnostic potential (Kamath et al., 2020). However, a marked increase in the number of antigens associated with Chagas disease has been observed when peptide microarrays were used (Carmona et al., 2015). These arrays, encoding overlapping peptides for 497 *T*. *cruzi* proteins lead to the identification of more than 2,000 epitopes and 97 novel antigens, corroborating previous observations that the immune response against this parasite is ample and directed toward a multitude of antigens. Considering that only a fraction of the predicted *T*. *cruzi* genes were studied, we wonder how many new antigens and epitopes would have been discovered if all parasitic genes were included in the array. gPhage display may potentially address these methodological shortcomings by displaying all possible peptides, hence epitopes encoded by the parasite genome.

Given that there are currently no available vaccines and patients have very limited therapeutic options (Marin-Neto et al., 2007; Cunha-Neto and Chevillard, 2014; Pérez-Molina and Molina, 2018), understanding the full repertoire of antigens may lead to the identification of biomarkers for improved disease outcomes. Upon acute infection, *T*. *cruzi* induces a strong antibody and T cell response that partially controls parasitism, subsequently leading to a low-grade, chronic infection. Over the course of several decades, up to one-third of infected individuals eventually develop cardiomyopathy (chronic Chagas disease cardiomyopathy; termed CCC), with patients dying of heart failure and/or cardiac arrhythmia; ∼60% remain asymptomatic and ∼10% develop digestive motility disorders (Bern, 2015; Pérez-Molina and Molina, 2018). Heart tissue damage is mainly due to chronic inflammation, which can be triggered either directly by the pathogen or by a host antigen cross-reactive immune response (Marin-Neto et al., 2007; Tarleton, 2001; Cunha-Neto and Chevillard, 2014; Bern, 2015; Pérez-Molina and Molina, 2018; Bonney et al., 2019). Notably, *T*. *cruzi* cross-reactive autoantibodies found in serum samples from patients with CCC recognize heart and nerve proteins, such as cardiac myosin, cholinergic and adrenergic receptors, and small ribonucleoproteins (Levin et al., 1993; Cunha-Neto et al., 1995; Kaplan et al., 1997; Giordanengo et al., 2000; Marin-Neto et al., 2007; Pérez-Molina and Molina, 2018; reviewed in Cunha-Neto et al., 2011). In addition, IgG isolated from patients with Chagas disease also induce heart anomalies in rats (Goin et al., 1997; López-Bergami et al., 2001). Given the strong and potentially detrimental host antibody response, determining the antigenic repertoire of Chagas disease will be required to understand its mechanistic role in disease development and progression. Strikingly, by using gPhage technology, here, we show that the immune antibody response against this single-cell parasite is ample and directed toward thousands of antigens, confirming the expression of hundreds of hypothetical proteins with functional domains, including members of multigene families.

## Results

### gPhage library construction

To design and develop gPhage, first we took advantage of the fact that most *T*. *cruzi* genes do not have introns and that almost half of the genome comprises coding sequences (El-Sayed et al., 2005). *T*. *cruzi* genomic DNA was sheared into small fragments and cloned into the phage display vector to produce a gPhage library containing ∼4 × 10^8^ transformants with inserts (Figure 1B). Sequencing of ∼2.2 × 10^5^ inserts showed that the vast majority were unique sequences, indicating the library had a high diversity (Figure 1C), with inserts having an average size of 143 bp (ranging from 9 to 423 bp insert size) (Figure 1D; Table S2; Data S3). Thus, the gPhage library generated here represented a ∼1,400-fold coverage of the parasite genome, displaying peptides with size up to 141 amino acid residues (average size of 48 amino acid residues) (Figure 1E; more details in STAR Methods). Trypanosomatids are unique regarding their mitochondrial DNA (kDNA), which is characterized by the presence of a network of concatenated circular DNAs of two types: minicircles and maxicircles (Lukeš et al., 2005). Therefore, we observed that while 92% of the inserts in the library were mapped to the parasitic genome, about half of the remaining inserts (64%) showed some degree of similarity to kDNA, indicating that they were also represented to some extent in the gPhage library.

Successful display requires that the insert be cloned in-frame with the 5′ and -3′ vector sequences (i.e., odds of 1 in 18 inserts), which encode the periplasmic export leader peptide and the reading frame for gene VIII, respectively (Figure 1F). Sequencing data from our gPhage library were generally in excellent agreement with these calculations, namely 17.8% of inserts yielded a full-length reading frame with the 5′ and 3’ sequences of the vector and nearly half of these peptides (48.3%) match a *T*. *cruzi* protein found in protein databanks; the remaining peptides correspond to alternative reading frames of genes or intergenic regions. Thus, while only 8.9% of phage clones in our gPhage library display a predicted *T*. *cruzi* protein (Figure 1G), we still have an approximate 100-fold coverage of the corresponding *T*. *cruzi* proteome (see calculations in STAR Methods, gPhage library coverage).

### gPhage library screening

**Figure 2:**
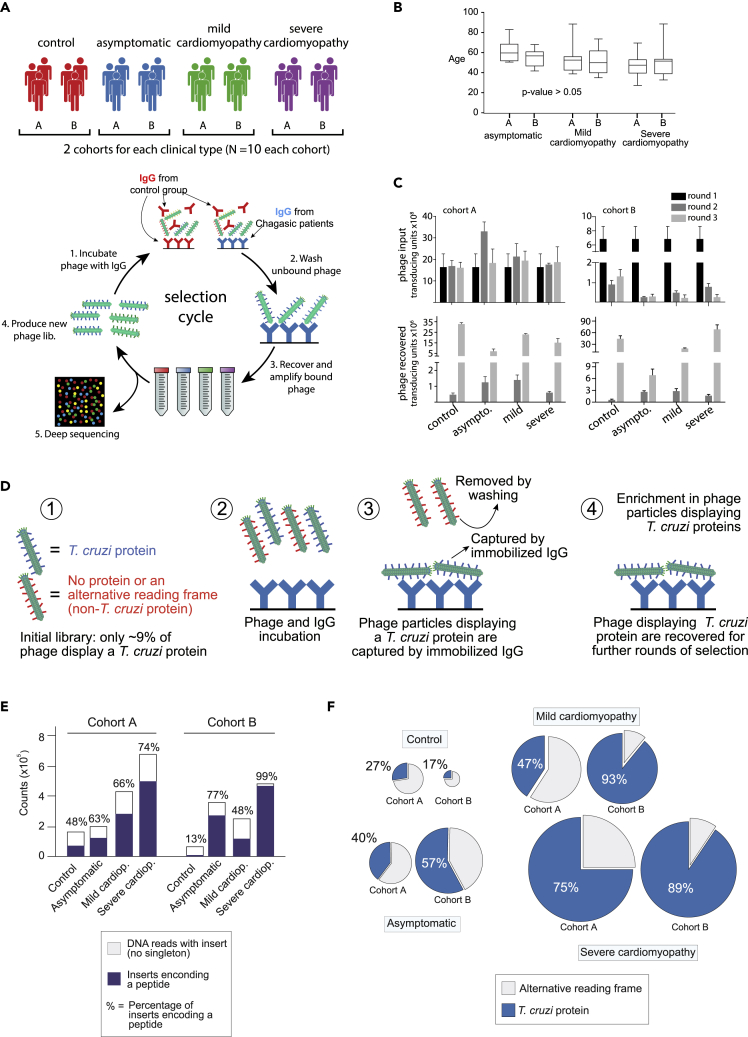
gPhage library screening (A) Scheme illustrating the cohort of patients and gPhage library biopanning strategy. (B) Box plot with the median age of patients with Chagas disease in each cohort (ANOVA); lines represent the median value, with box extending from the 25th to 75th percentiles and whisker from minimum to maximum values. (C) Graphs representing phage input (top) and phage recovered (bottom) during each round of the biopanning; bars indicate average values between three measurements and error bars indicate SEM (standard error of the mean). (D) Scheme illustrating a successful biopanning. Only phage particles displaying a *T*. *cruzi*-derived peptide would be capture by the immobilized IgG from patients with Chagas disease. The remaining phage particles are washed away and lost during the successive rounds of biopaning. (E) Phage recovered from the third round was submitted to large-scale sequencing and the bioinformatic pipeline. The bar graph indicates the number of unique reads containing a DNA insert (the sum of white and purple bars) and the number of DNA inserts that encode a peptide (purple bar). (F) Pie chart indicating the percentage of peptides (purple bar in E) that match a *T*. *cruzi* protein (blue) or correspond to an alternative reading frame of the DNA insert (white).

To identify the antigens and epitopes recognized by the serum samples of patients with Chagas disease, we incubated the gPhage library with purified immunoglobulin G (IgG) from patients at different stages of the disease (asymptomatic, mild CCC, or severe CCC). For each stage of the disease, IgG from individual patients was combined into pools to produce two biological replicas (named cohorts A and B; N = 10 patients in each cohort) (Figure 2A). Asymptomatic patients were volunteer blood donors who have incidentally tested positive for Chagas disease in two consecutive screening assays but showed no signs of cardiac abnormalities during the electrocardiographical and echocardiographical evaluations. Thus, patients with CCC showed electrocardiogram or echocardiogram alterations typical of the disease and were classified as mild and severe based on the echocardiographic measurement of left ventricular ejection fraction (LVEF), with patients with severe CCC having an LVEF of 40% or lower (STAR Methods and Table S1). The median age of patients in each group varied from 48 to 60 years but there were no significant differences in age distribution among the individual cohorts (*p* value > 0.05) (Figure 2B). IgG from volunteer blood donors who screened negative for Chagas disease served as a negative control. To minimize selection of ubiquitous antigens recognized by the general population, the gPhage library was also incubated with soluble IgG pooled from the control group to compete with immobilized IgG from patients (Figure 2A) (Faulin et al., 2019). After 3 rounds of selection, we observed phage enrichment for all groups (Figure 2C), and all samples from the last round were sequenced with an average of 672 thousand reads per sample (minimum = 449,000 and maximum = 1,130,000 reads) (Table S2). Sequences had to be present in at least two individual reads to be considered valid (Dias-Neto et al., 2009), a requirement that removed singletons (4–30% of reads), reduced sequencing errors, and increased our confidence in the final list of candidate-displayed antigens.

**Figure 3:**
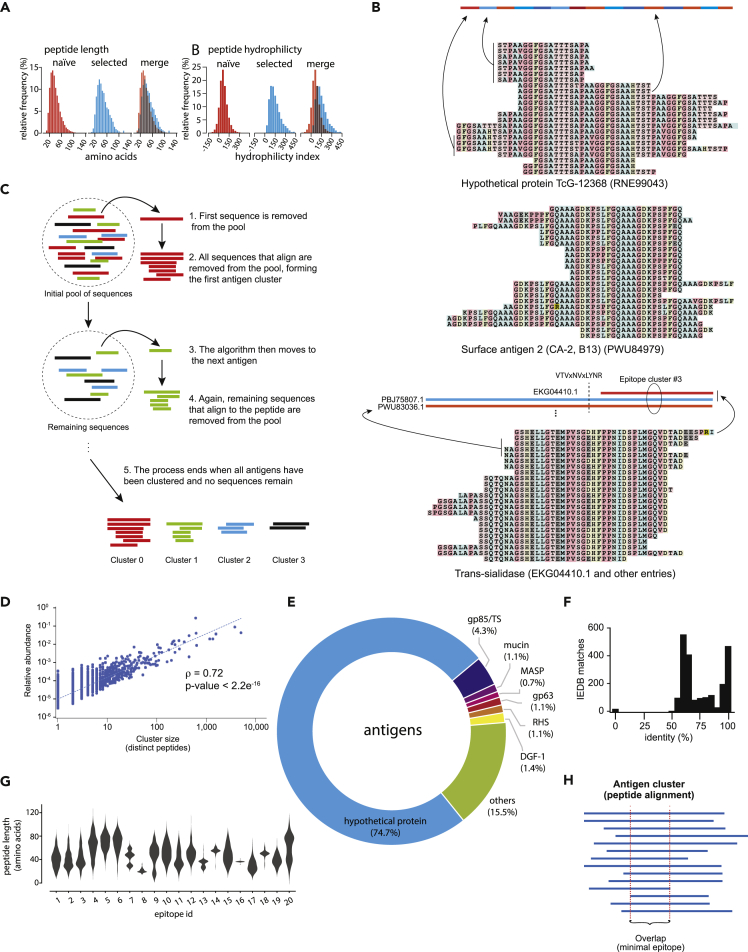
Antigen identification and epitope mapping (A) The plots show peptide distribution as per length (left) and hydrophilicity (right) in the naive library (red) and after three rounds of selection with IgG (blue) of patients with Chagas disease (B) Alignment illustrating three clusters of epitopes. Each line represents a unique *T*. *cruzi*-derived peptide identified in the final round of selection. The top two clusters comprised epitopes belonging to repetitive sequences of the same protein. The bottom one is a cluster that maps to an epitope shared by distinct members of the gp85/trans-sialidase multigene family. (C) Scheme to illustrate the algorithm used to cluster all unique sequences identified in the third round of the biopanning to identify the 3,964 epitope/antigens pairs. (D) Scatchard plot showing the correlation between antigen cluster abundance x number of peptides in the cluster (Spearman correlation). (E) Pie chart displaying all final antigens and their corresponding similarity to *T*. *cruzi* protein families. (F) Bar graph indicating the number of antigen clusters that match previously described *T*. *cruzi* antigens (IEDB). (G) Violin plot displaying the length and density of selected clusters of antigens. (H) Cartoon illustrating our epitope mapping strategy. After clusterization, all sequences were aligned and the minimal overlapping region shared by all peptides was identified.

While most phage particles in our initial gPhage library (82.2%) did not display any peptides (out-of-frame inserts) and only 8.9% displayed *T*. *cruzi*-derived peptides, upon selection, there was considerable enrichment of particles displaying a peptide (except for control cohort B) (Figures 2D and 2E). For both control groups, the increase did not necessarily translate into the display of *T*. *cruzi*-derived peptides, as most of the peptides were alternative reading frames of the corresponding parasite genome (Figure 2F). Conversely, for patients with Chagas disease, selection did result in enrichment of *T*. *cruzi*-derived peptides, with patients with severe CCC showing the highest level of display (75% and 89%, for cohorts A and B, respectively) (Figure 2F). Asymptomatic patients yielded phage recovery and percentage of inserts in the correct reading frame somewhere between CCC and control groups (40% and 57%, for cohorts A and B, respectively). There was no significant change in peptide size compared with the unselected parental library, although there was an increase in hydrophilicity (Figure 3A), as expected, given that antibody epitopes are usually water-exposed. Thus, our selection process led to an enrichment in *T*. *cruzi* antigens recognized by IgG from patients with Chagas disease.

### Epitope identification

Overall, we have identified a total of 30,430 unique phage inserts encoding *T*. *cruzi*-derived antigens (16,203 in cohort A and 14,227 in cohort B). However, most of them represent fragments from the same protein with minor variations in length or in amino acid sequence, presumably owing to the high number of multigene families and repetitive elements (Figure 3B). To generate a nonredundant list of epitopes, we developed an in-house software application to binning antigen sequences (Data S3). Peptides were sorted by size (shorter to longer), and the first peptide antigen (so-called seed) was then removed from the list and compared (aligned) with the remaining peptide sequences. Those that matched the seed peptide antigen (at least 80% identity) were also removed from the list and binned to form the first cluster of antigens. This binning process was repeated until there was no peptide left in the list (Figure 3C), generating 1,548 clusters of epitopes (minimum = 2; maximum = 4,794 peptide fragments); the remaining 2,416 identified epitopes were single-peptide sequences, yielding a total nonredundant list of 3,964 epitopes (Data S1). During the binning process, a consensus sequence was also extracted for the 1,548 clusters of epitopes. In sum, we have identified 1,548 unique epitopes that were defined by clusters of at least 2 or more distinct peptides with a further 2,416 epitopes that were defined by a single peptide. The epitopes varied in length with a median size of 39 amino acid residues (minimum = 13 and maximum = 119). We also observed a clear difference between our biological duplicates: the selection process was more stringent for cohort B, which resulted in one order of magnitude smaller number of identified epitopes (N = 315), compared with cohort A (N = 3,871). This could be owing to differences in phage input, which was lower for cohort B selection (Figure 2C). However, 70% of all epitopes from the cohort B were contained within cohort A, therefore confirming the reliability of the selection process used here. Next, we performed protein database search and associated each epitope with a T. *cruzi* protein (antigen) (Data S1).

Epitopes were subsequently quantified in two ways: (1) the number of fragments or distinct peptides in each cluster and (2) their overall abundance (percentage of reads that aligned to the parasitic genome - [reads associated with the epitopes]/[total reads in the sample that align to the genome] x 100) (Data S1; and all clusters, alignments, and consensus sequences are provided Data S2). We observed a significant correlation between these two quantification indexes (Spearman correlation ρ = 0.72, *p* value < 2.2 × 10^−16^): the larger clusters were also the most abundant, strongly suggesting that both values could be used as proxies for immunogenicity (Figure 3D).

### Validation and epitope mapping

To validate the identified candidate epitope, we next performed online searches of the Immune Epitope Database and Analysis Resource (IEDB; www.iedb.org) for known Chagas-disease-associated antigens. All Chagas disease antigens and epitopes were downloaded from the IEDB database and compared with each individual peptide antigen identified in our study by using a pairwise sequence alignment algorithm. We observed that 469 (21%) of the epitopes were identical to epitopes in our working data set, including B13, gp85/trans-sialidases, mucin, and SAPA, among others (Figure 3E). Given the large number of multigene families, repetitive elements, and genomic variability among the several parasite strains, we have relaxed our stringency and expanded our analysis to include similar as well as identical antigens. Thus, of all Chagas disease epitopes in the IEDB, 2,029 epitopes (93%) share at least 60% identity with epitopes in our list (Figure 3F). In sum, we observed an excellent agreement between our set of antigens and previously described *T*. *cruzi* epitopes reported in the literature and/or found in the IEDB online database. Remarkably, our data set also yielded a large number of previously undescribed antigens/epitopes – 49% of identified epitopes in this study (1,935 of 4,964) were not associated with any epitopes in the IEDB. Almost three-quarters of all epitopes identified (74.5%) with gPhage belonged to hypothetical proteins (Figure 3F), implying they might perhaps be expressed during the infection cycle of *T*. *cruzi*.

Given the need for biomarkers of disease progression or cure, we next selected epitopes for validation by using ELISA that were specific for a particular cohort of patients (asymptomatic, mild, or severe CCC), including epitopes belonging to hypothetical proteins EKG03457, PWU85340, ESS63713, and PWU83017 and protein phosphatase 2C, a putative RNA-binding protein (Table 1). Epitopes found in newly described and highly abundant antigens from large clusters and tandem-repetitive elements were also selected for validation (hypothetical protein RNE99043 and PBJ77896; microtubule-associated protein RNF14378). Because many of these epitopes were defined by large peptides, we performed epitope mapping to facilitate design of suitable synthetic peptides (∼15–29 amino acid residues long) for ELISA. We used the overlap among the distinct peptide fragments in the larger clusters to search for consensus and/or minimal sequences that could represent epitopes recognized by *T*. *cruzi*-reactive IgG in the sera of patients with Chagas disease. In most clusters, there was substantial variability in peptide length and their overall overlap regions (Figure 3G). We used the information to extract the minimal intercept region among all peptide fragments in the cluster (Figure 3H). In some cases, these regions corresponded to tandem repeats, which were further reduced to discrete units to design a final list of 20 peptides for experimental validation (Table 1). Only two of our selected epitopes contained minimal consensus sequences longer than 30 amino acid residues, which were then split into shorter overlapping peptides.

**Table 1:** Epitopes selected for validation (ELISA)

Cluster #	Synthetic peptide sequence	Antigen (genbank ID)	Validation	Selection criteria	Number of peptides in the cluster	Abundancy (%)^a^
0	GGFGSATTTSTPAAGGFGSAAHTSTPAVG	Hypothetical protein (RNE99043)	ELISA (+)	Mild cardiopathic	4794	14
1	FGQAAAGDKPPLFGQAAAGDKPSL	Surface antigen 2 (PWU84979)	ELISA (+)	Asymptomatic and mild cardiopathic	3560	15(A)/8.1 (MC)
2	YKRALPQEEEEDVGPRHVDPDHFRSTT	microtubule-associated protein (RNF14378)	ELISA (+)	Asymptomatic and mild cardiopathic	1481	2.9 (A)/8.5 (MC)
3	NAGSHELLGTEMPVSGEHFPPNIDSPL	Trans-sialidase (EKG04410)	(−)	Asymptomic & mild cardiopathic	1300	1.1 (A)/4.8 (MC)
7	PPHTRRVTVRCGPPSCADE	Hypothetical protein (PBJ77896)	Phage-ELISA (+)	Severe cardiopathic	587	40/82
8	PSPSAAQHSLPRRHPSPSAAQHSLPRRH	Hypothetical protein (PWV06054)	(−)	Control	431	1.5/0.5
9	KVAEAEKQRAAEATKVAEAEKQRAA	Flagellar repetitive antigen (AAA30177)	ELISA (+)	Tandem repeats and mild cardiopathic	427	3.8
10	APAKAAAAPAKAAAAPAKAAAAPA	60S ribosomal protein L19 (PBJ71794)	ELISA (+)	Tandem repeats and asymptomatic	346	1.2/1.0
12	AAKTAMGEAGGRSWSNVVKSPHSPR	Hypothetical protein (EKG07867)	ELISA (+)	Mild cardiopathic	271	4.4
15-2^a^	PCRGLNYRFPVECGDVLFLGSDGVF	Protein phosphatase 2C (RNF23118)	ELISA (+)	Asymptomatic	217	4.2
16	PAAKPAAKPAAKAPAPKA	Putative RNA binding protein (EKF98911)	ELISA (+)	Mild cardiopathic	202	1.9
19	SSLAGSDGVGLAGGASSIESFEGLP	Hypothetical protein (EKG03457)	ELISA (+)	Asymptomatic	168	1.1
24	AATEKRQSVNNYTTPGDSDGSTAVS	MASP (PBJ79554)	ELISA (+)	Mild cardiopathic	106	1.6
26	IEEVRGAAPLGKYALVNTLEGSDG	Hypothetical protein (RNE99359)	(−)	Asymptomatic	100	2.5
32-3^a^	PEQEKIPEMVSLIFFGSDGVNLEGT	Trans-sialidase (PWU85340)	ELISA (+)	Asymptomatic	84	0.7
48-3^a^	TSFGSDNEKLAGSEGIFGSTSSFST	Hypothetical protein (ESS63713)	ELISA (+)	Asymptomatic	53	0.3
49	LADELEQKAAENERLADELEQKAAENER	Flagellar attachment zone protein 1 (PWU93573)	(−)	Tandem repeats and mild cardiopathic	51	0.02
53	YIDGKSLGEEEVPLTGEKPLELF	Trans-sialidase (PBJ76118)	ELISA (+)	Mild cardiopathic	45	0.5
92	ELQEERRAVARAEVELKKRLQ	Hypothetical protein (PWU83017)	(−)	Severe cardiopathic	25	0.4
233	PHGTQRRVATRVEAV	Phospholipase A2-like (EKF31673)	(−)	Severe cardiopathic	10	0.4
415	ERRQLLERDPRRNAREIAALEESMN	Calpain-like cysteine peptidase (PBJ79197)	ELISA (+)	Mild cardiopathic	6	10^−5^
546	PAEDVQELVAPAEDVQELVAPAEDV	Hypothetical protein (PWU87792)	(−)	Tandem repeats and severe cardiopathic	5	10^−5^

Synthetic peptides were designed for 20 antigens selected based on their frequency among the different groups of patients and number of peptides in the cluster (A = asymptomatic; MC = mild cardiopathic patient).

a Overlapping peptides were designed and tested. Only reactive peptides are shown.

**Figure 4:**
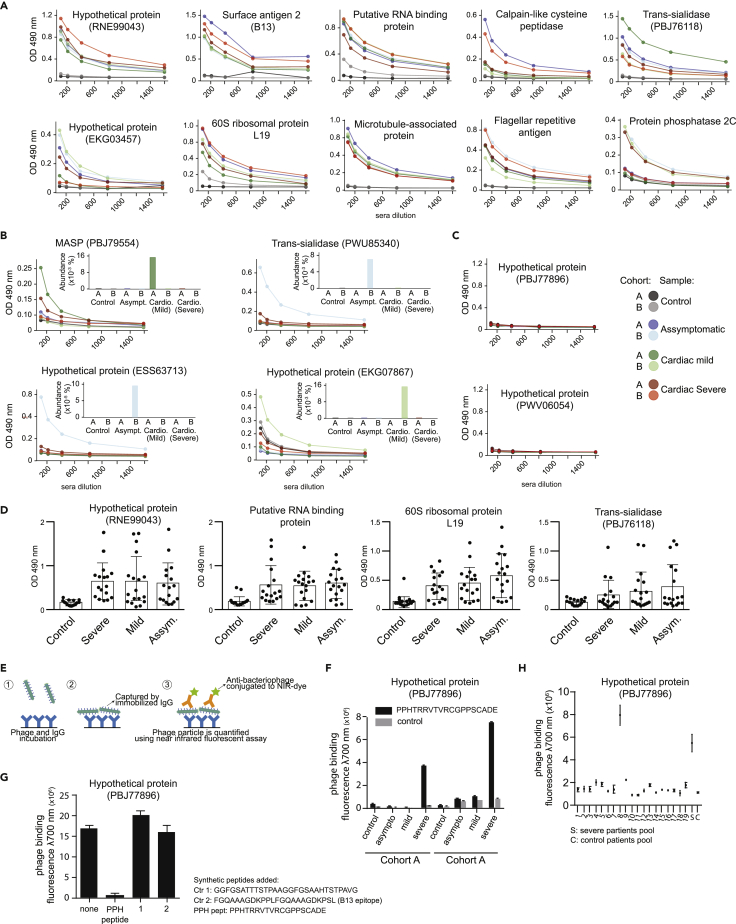
Antigen validation (A–C) ELISA reactivity of each pool of IgG from both cohorts with synthetic peptides representative of selected clusters of antigens. The insert bar graph (B) shows the frequency of the corresponding antigen in each group of patient and cohort. (C) Peptides that were not recognized by the pool of IgGs. (D) Scatterplot of ELISA reactivity of IgG (d1/200) from individual patients with selected synthetic peptides (bars indicate average values; error bars indicate SEM). (E and F) (E) Scheme to illustrate the phage ELISA assay and (F) reactivity of phage PPHTRRVTVRCGPPSCADE with each pool of IgG from both cohorts. (G) Phage ELISA competition assay in the presence of absence of synthetic peptides (H) Reactivity by phage ELISA of phage PPHTRRVTVRCGPPSCADE with IgG from individual patients from the cardiomyopathic group (severe). All values represent the average of three measurements and error bars indicate the SEM (F–H).

We validated our findings by ELISA against the pool of IgG from each patient group. We observed that 13 of the 20 peptides (65%) were recognized by IgG antibodies in ELISA: 9 were recognized by all patient groups, in addition to the immunodominant epitope B13 (surface antigen 2, positive control), which reacted strongly with the IgG pools (Figure 4A). Four peptides showed a specific response toward the pool of IgG for which they were selected and had shown the highest abundance (in percentage) in the selection (Figure 4B). The remaining 7 peptides did not react with the pools of IgG in the ELISA tests (Figures 4C and S1), including one peptide that was designed based on an antigen that was enriched in the control group (hypothetical protein PWV06054). We tested the reactivity of individual patient serum samples toward epitopes recognized by IgG pools, corroborating previous observations that the anti-*T*. *cruzi* antibody response of infected patients is heterogeneous and directed toward a multitude of antigens (Figure 4D). Collectively, these experimental results validate gPhage as robust technology for epitopes/antigen identification.

### An immunodominant conformational epitope

The lack of ELISA reactivity of some synthetic peptides could be owing to low binding capacity of this specific peptide or to loss of conformational properties upon immobilization on the assay plate. Phage particles encoding a proline-rich 19-residue peptide PPHTRRVTVRCGPPSCADE, derived from hypothetical protein (PBJ77896), were recognized specifically by IgG from the two pools of patients with severe cardiomyopathy; however, the immobilized plastic-bound synthetic peptide was not recognized in ELISA (Figure 4C). This peptide was designed based on a large antigenic cluster (# 7) containing 587 unique peptides (the abundance for this cluster was 40% and 82% in pooled cohorts A and B, respectively), with the peptide sequence being identical to the most frequent phage sequence in the cluster. To investigate the lack of ELISA reactivity to the peptide, we isolated the phage-displayed peptide and tested its reactivity by using a phage-based assay (Figure 4E). We observed that the phage-displayed peptide PPHTRRVTVRCGPPSCADE is specifically captured by the pool of IgG derived from patients with severe cardiomyopathy of both cohorts (Figure 4F). Phage PPHTRRVTVRCGPPSCADE was selected because it was the most abundant phage recovered in the biopanning. Finally, targeted phage binding was specific and inhibited by the synthetic peptide PPHTRRVTVRCGPPSCADE but not by two other control peptides, both *T*. *cruzi* antigens (B13 and cluster #0) (Figure 4G).

We tested the binding of PPHTRRVTVRCGPPSCADE phage to IgG from each individual patient in the pools (cohort A and B) and found that it bound to IgG from a single patient (#8), which had been inadvertently added to both pools of IgG from patients with severe CCC (Figure 4H). Again, IgG from this single patient failed to react with the immobilized peptide confirming that the lack of ELISA reactivity was not due to a dilution effect. Thus, gPhage screening is capable of detecting patient-specific immunodominant epitopes within an admixture of IgG from several individuals. These results also indicate that gPhage allows for mapping hard to identify epitopes, either owing to lack of binding to plastic or the presence of a conformational epitope, which would be challenging to identify with standard biochemical assays (such as ELISA and/or peptide microarrays). In sum, we have validated 14 antigens/epitopes that are reactive with serum from patients with Chagas disease, representing a success rate of at least 70% (14 of 20 gPhage-identified antigens), thereby validating gPhage as an effective technology for epitope mapping in this setting.

### The Chagas disease antibody response

We used our data to analyze the extent of Chagas disease antibody response. Of the epitopes we identified, 74.7% are hypothetical proteins (Figure 3E), and among the largest clusters and most abundant antigens, there are several that contain tandem repetitive sequences (Table S3), indeed a common feature among immunodominant protozoan antigens (Ibañez et al., 1988; Goto et al., 2008; Goto et al., 2010; Valiente-Gabioud et al., 2011). In fact, the *T*. *cruzi* genome contains a high number of repetitive elements and multigene families, which seem to play a role in infection (De Pablos and Osuna, 2012). Of the nonhypothetical antigens, half belong to members of these multigene families, with the gp85/trans-sialidases being the most prevalent (4.3% of all known antigens identified) (Figure 3E). Parasitic antigens belonging to the disperse gene family, gp63, mucin, RHS, and MASP multigene families were also identified, further confirming the usefulness of the methodology.

**Figure 5:**
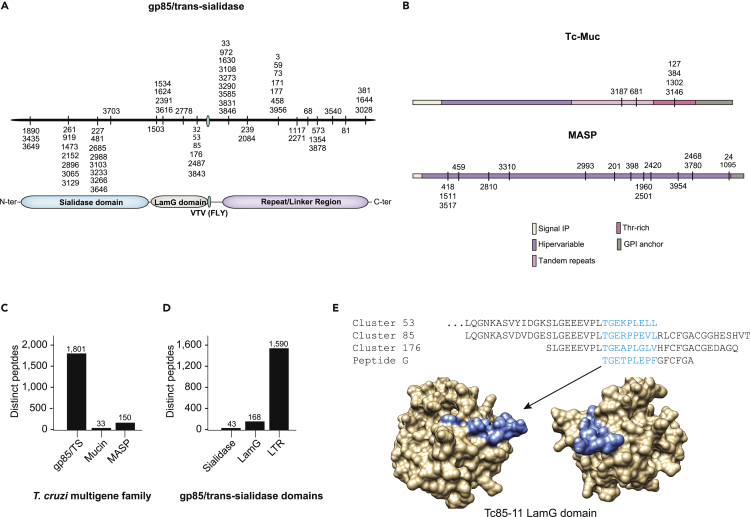
The Chagas disease antibody response (A and B) Mapping of individual clusters of antigens identified in this study to the gp85/trans-sialidase (A), mucin, and MASP (B) families of proteins. (C and D) Bar graphs indicting the total number of unique peptide sequences that map to each gp85/trans-sialidase domain (C) and each multigene family (D). (E) Alignment for gp85/trans-sialidase clusters 53, 85, and 176 with a previously described epitope (peptide G) for a neutralizing antibody; surface representation for the LamG domain of the *T*. *cruzi* Tc85-11 protein. The location of peptide G is indicated in blue.

Given we mapped epitopes to proteins belonging to multigene families and many of the antigenic clusters comprise multiple peptides (at least 1 and up to 4,794 unique sequences), we used the numbers of unique sequences in each cluster as proxies for antigenicity. We reasoned that the different sequences within the individual clusters likely represent variants of the same epitope shared by different member of each multigene family. In total, 60, 6, and 17 clusters of epitopes could be unequivocally mapped to members of the gp85/trans-sialidase, mucin, and MASP families, respectively (Figures 5A and 5B). Summing up the number of sequences in each cluster revealed a total of 1,801, 33, and 150 unique sequences for the gp85/trans-sialidase, mucin, and MASP multigene families, respectively (Figure 5C). The gp85/trans-sialidases and MASP families have similar numbers of genes in the *T*. *cruzi* genome (and mucins about half this number) (El-Sayed et al., 2005), data suggesting that the gp85/trans-sialidases represent the most antigenic multigene family.

The antibody response toward mucin and MASP proteins was preferentially directed toward their hypervariable and tandemly repetitive domains (Figure 5B). For the MASP family, most of the epitopes (108 of 150; 71%) were directed to the C-terminal domain which contains the GPI-anchoring site (De Pablos et al., 2016; Durante et al., 2017). Interestingly, the largest cluster (#24, containing 106 peptides; 70% of the MASP epitopes) matches very precisely to the very C-terminal tip of the protein, suggesting that the immunodominant epitope does not include the sorting signal (GPI additional sequence, or SP), but it is directed to the mature C-terminal region, as proposed (Durante et al., 2017). Most of the gp85/trans-sialidase epitopes were also directed toward the carboxy-terminal domain (1,591 of 1,801; 88%), with only a minority mapping to two regions involved in infection: the sialidase domain (43 of 1,801; 2.4%), associated with trans-sialidase activity and cell binding (Colli, 1993; Alves et al., 1986; Giordano et al., 1999; De Pablos and Osuna., 2012; Mattos et al., 2014; Freire-de-Lima et al., 2015), and the LamG domain (182 of 1,801, 10%), involved in cell infection through interaction with intermediate filament proteins (i.e., cytokeratin) (Figure 5D) (Teixeira et al., 2015). At least three epitopes that map to the LamG domain (clusters #53, 85, and 176) overlap with peptide G, a previously described epitope for the *T*. *cruzi*-neutralizing monoclonal antibody (H1A10) that recognizes a subset of the gp85/trans-sialidase family (Alves et al., 1986; Giordano et al., 1999) (Figure 5E).

**Figure 6:**
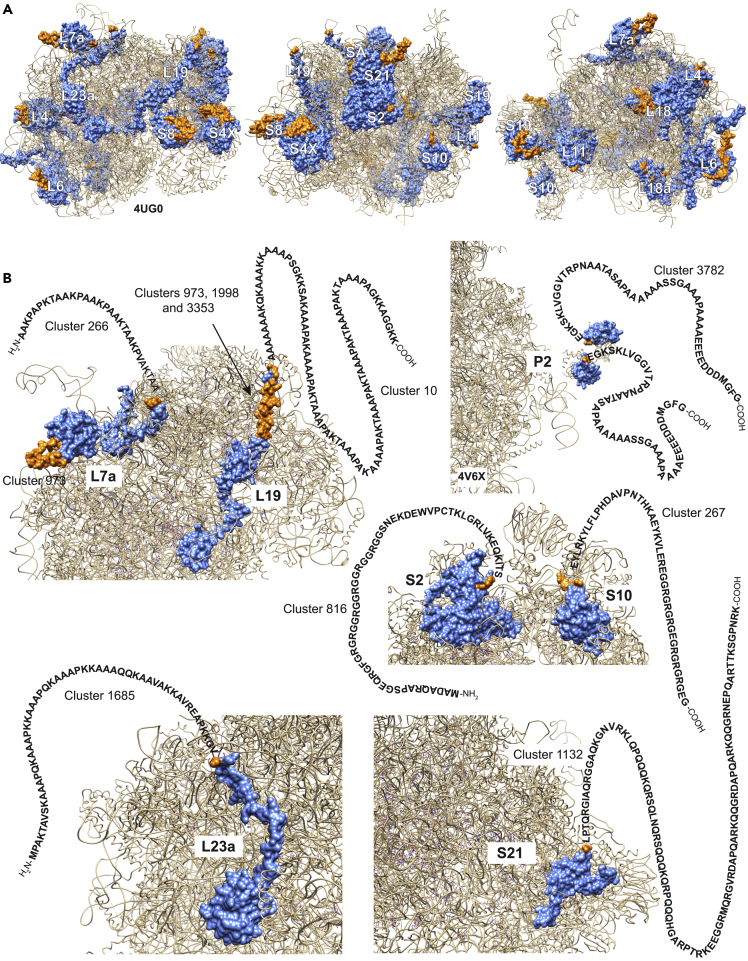
*T*. *cruzi* ribosome (A) Clusters of antigens that match *T*. *cruzi* ribosomal proteins were alignment to proteins (blue) from the human ribosome (PDB #4UG0 and 4V6X—for the P2 protein). Their 3-dimensional position is indicated (orange). (B) Most of the clusters represent the insertion of long amino- or carboxy-terminal peptides containing repetitive elements.

A common feature of the immune response to *T*. *cruzi* is the presence of antigens containing tandemly repetitive elements (Table S3). We identified a number of these elements in domains of proteins highly conserved in evolution: 21 antigen clusters (most of them containing repeats) mapped to epitopes belonging to 17 distinct ribosomal proteins. Ribosomal proteins elicit the production of high levels of autoantibodies in CCC sera and have classically been implicated in the pathogenesis of Chagas disease (Levin et al., 1993; López-Bergami et al., 2001; Motrán et al., 2000; Kim et al., 2019). Given the available structural data for the eukaryotic ribosome, we used these epitopes as a proof-of-concept to show that gPhage is an effective tool to further refine epitope mapping within 3D structures. Ribosomal proteins are highly conserved across species, so we could unequivocally align the 17 *T*. *cruzi* epitopes to their human orthologs and map their equivalent position in space by using the human ribosome as a model. We observed that the *T*. *cruzi* ribosome is highly “decorated” with these repetitive elements, most of them being surface-exposed epitopes (Figure 6A). Only two antigenic clusters (#1532 and #1775 that map to proteins L18 and L18a, respectively) were buried inside the ribosome (cryptic epitopes). The remaining clusters mapped to surface loops on the ribosome, such as cluster 972 in protein L7a and most seem to be large tandem repeats found in the amino- or carboxy-terminal domains of these proteins (Figure 6B). Thus, the data generated by the gPhage platform combined with structural data allow marked refinement of large-scale epitope mapping.

## Discussion

Here, we showed that gPhage provides an unbiased approach for the simultaneous identification of antigens and epitopes in the analysis of antibody repertoires associated with parasitic diseases. gPhage can also serve for the rapid identification of antigens and antibody epitopes of pathogens from either emerging/reemerging or neglected infectious diseases. An advantage of gPhage is the size of the libraries that can be generated, which often contain billions of inserts (Michaloski *et al*, 2016; Beppler *et al.*, 2016; Tang *et al.*, 2019) and which can outperform arrays limited by the number of peptides and proteins that can be synthesized and inserted into the array (usually, millions or less). The gPhage methodology can be used with nothing more than the annotated genome of the study organism to serve as a guide during the analysis and even this is not an absolute requirement, making gPhage a cheaper and easier alternative for antigen identification than other existing methods. gPhage can be a useful tool for antigen discovery: by using genomic DNA, rather than cDNA, biases due to gene expression can be avoided, and genes can be sampled that would otherwise be missed if they have not yet been identified or are only expressed by the parasite at specific stages of the disease. Our data provide compelling evidence for the expression of many currently hypothetical proteins in the *T*. *cruzi* genome during the infection process, increasing the number of putative molecular targets for the development of drugs and vaccines for the disease. One possible limitation of gPhage is that peptides toxic to the bacterial cell or the bacteriophage particle, including peptides with structural constrains for proper display are probably underrepresented in the library. Nevertheless, the gPhage library used in this study encodes more than 1,400 times the genome of the parasite, resulting in multiple overlapping peptides likely covering the entire proteome. This should mitigate problems regarding structural constrains and, perhaps, toxicity because there are several alternative versions of the same epitope.

Although we envision gPhage to be used even with organisms that do not have genome information, if available, it is always a good strategy to build the libraries using gDNA from strains with curated genome data. When we began the study, we elected the Sylvio X10/1 strain because it was the only no-hybrid strain with genome data available. Building a gPhage library from a hybrid strain would require twice the number of transformants, which we were unsure would be feasible. Given that our library covers more than 1,400 times the genome of a nonhybrid strain or the equivalent to at least 700 times the genome of hybrid strain, we believe now that any *T*. *cruzi* strain could be used for gPhage. Considering the recent advances in sequencing technology (long-read sequencing) that have helped to close this gap and to produce better annotated genomes (Berná *et al.*, 2018), usage of strains with better annotated genomes (such as TTC – hybrid and DM28c – nonhybrid) would allow for more precise antigen identification pipelines. Nevertheless, despite the limitations of the Sylvio X10/1 genome data, we have assigned most of the 3,964 epitopes to annotated *T*. *cruzi* proteins by complementing the pipeline with data from other better-annotated genomes.

Still, only half of the genes in trypanosomatid genomes have been assigned to proteins with known function and a significant percentage of these hypothetical genes are unique to trypanosomatid parasites or species-specific genes (El-Sayed *et al.*, 2005). Thus, it is not surprising that a small percentage of the epitopes we identified have shown very low similarity to known proteins, given the challenges to annotate these hypothetical genes, such as lack of orthologs to assist with the annotation. These unmapped epitopes may belong to proteins that have not been annotated, yet. They could also represent spurious peptides encoded by alternative reading frames of the parasite's genome that resulted in mimotopes specifically recognized by the IgG of patients with Chagas disease. The presence of several multigene families presents another difficulty for correct genome annotation, and it also influences our antigen assignment pipeline. We noticed that several epitopes showed significant similarity to proteins annotated as belonging to one of these multigene families, such as the gp85/trans-sialidase, mucin, or MASP families. However, some of these antigens lacked the characteristic elements of their corresponding family (i.e., sialidase domain, Thr repeats) or encode truncated versions of these proteins. Therefore, a separate pipeline was necessary to correctly assign and curate epitopes belonging to the gp85/trans-sialidase, mucin, or MASP families (Figure 5) (the 3,964 epitopes were individually compared by pairwise alignment to representative members of these families – see STAR Methods for details). In summary, the antigens assigned in our final table should be used as guideline and with caution. For more detailed studies, we recommend that each individual epitope should be curated with an appropriate computer pipeline to determine more accurately to which *T*. *cruzi* antigen it belongs.

Considering that this is a resource data set, despite its limitations, we have opted to include all epitopes in our final list. To assist researcher in the field to use and analyze the data set, we have also incorporated the results of the database comparison (description and accession code for the top hit, along with the expected value), the number of unique peptides that compose the epitope cluster, and their frequency in each cohort of patients used for the biopanning (Data S1). For instance, high expected values from the pairwise alignment may indicate poor antigen identification. The alignments for all clusters and consensus sequences are also provided and they may be helpful for epitope mapping (Data S2).

We have also included the quantification of all epitopes using two criteria: their overall abundance in the biopanning and the number of unique peptides that comprise the antigenic cluster, which showed good linear correlation. It is important to note, however, that quantification of individual antigens/epitopes based on their abundance might be distorted by the immune response of individual patients when IgG from several patients are combined during the selection process, as it was carried out here (cohorts of 10 patients). For instance, IgG from patient #8 (present in both cohorts of severe CCC) capture in large numbers of phage displaying peptides belonging the same epitope: peptides belonging to cluster #7 (consensus sequence PPHTRRVTVRCGPPSCADERA) correspond to 40% and 81% of all recovered phage in both cohorts A and B of severe CCC, respectively. It means that epitopes recognized by IgG from the other 9 patients were squeezed into the remaining 60% and 19% of the recovered phage for each cohort A and B, respectively. We did not observe other clusters with such high abundance in the remaining cohorts (asymptomatic and mild CCC), so this problem should be limited to patients with severe CCC. To avoid this bias in future studies, we recommend that gPhage selection should be performed using IgG of individual patients and not pool of patients.

Nevertheless, 1,548 clusters have at least two unique peptides (1,519 of 3,871 epitopes [39%] for cohort A and 214 of 315 epitopes [69%] for cohort B) and may be a better indication of a true epitope compared with the remaining 2,476 single-peptide epitopes. Certainly, because phage selection using patients’ IgG cohort A was less stringent than with cohort B, a significant proportion of the former epitopes are represented by low-abundance single peptides. But, it is also difficult to assess whether the selection with cohort B was actually too stringent to the point of missing epitopes, enriching in immunodominant epitopes. Here, we validated at least one epitope (cluster #415, calpain-like peptide—Figure 4A) belonging to a low-abundance cluster (∼10^−5^%), which is not far from the lowest-abundance clusters (∼10^−6^%), indicating that other low-frequency epitopes might also be true antigens. Again, whether all these low-abundance epitopes are genuine antigens or not, they have all been include in the final data set along with their abundance in each cohort. For instance, there are 209 epitopes in our list which are recognized only by the control IgG, all low-abundance epitopes (10^−6^ to 10^−5^%) represented by a single peptide (only 4 of them are clusters with 2–4 peptides). They may represent cross-reactive *T*. *cruzi* epitopes (or mimotopes) recognized by natural antibodies present in the general population. We opted to do this rather than set up specific cutoff values, such as abundance or number of peptides that compose the epitope cluster. The reason for that is because at least some of the low-abundance epitopes correspond to previously validated epitopes such the MASP immunodominant C-terminal epitope (cluster #1095, with 2 peptides) (De Pablos et al., 2016; Durante et al., 2017) or antigen R13 (cluster #3782, single peptide) (Motrán et al., 2000; Zrein et al., 2018).

As a proof-of-principle, we performed two large-scale epitope mapping studies with this data set to illustrate how the gPhage platform can be used to build a comprehensive atlas of the antibody response of a large cohort of patients and, critically, pinpoint many of the epitopes from these antigens to specific protein regions/domains. The strong antibody response of patients with Chagas disease toward the carboxy-terminal domain of members of gp85/trans-sialidase multigene family seems to protect the parasite by preventing the production of high-titer neutralizing antibodies against the other two functional domains (sialidase and LamG), which have been implicated in cell infection (Alves et al., 1986; Giordano *et al.*, 1999; Teixeira *et al.*, 2015). As explained previously, because we performed the biopanning using pools of IgG, epitope quantification might have been influenced by individual patients with high-titer IgG (such as patient #8 in the severe-CCC groups). However, overall, our results agree with previous observations that cross-reactive epitopes distributed throughout the gp85/trans-sialidase family, and strong immunodominant epitopes such as the repetitive shed acute-phase antigen, located in the carboxy-terminal domain, are important to prevent buildup of a strong humoral response against the sialidase domain (Alvarez *et al.*, 2001; Pitcovsky *et al.*, 2002). Finally, our analysis of the immune response toward the *T*. *cruzi* ribosome illustrates how the combination of antigen identification and epitope mapping can provide insight into protein domains and/or their 3-dimensional structure.

Ribosomes are a common element in the immune response in many parasitic and microbial infections (Normier et al., 2007; Ramos *et al.*, 2009; Valencia-Hernandez *et al.*, 2020). In *T*. *cruzi*, cross-reactive antibodies against epitopes present in ribosomal protein P1 and P2 (antigen R13), which map to cluster #3782, have been implicated in cardiomyopathy (Motrán et al., 2000) or used as biomarkers (Zrein *et al.*, 2018). It is as yet unknown whether the long-tandem repeats found in *T*. *cruzi* ribosome proteins play a role in protein synthesis. Given their locations—protruding from the surface of the ribosome particle—and their lack of conservation within otherwise evolutionarily highly conserved ribosomal proteins, one might speculate that their function may be, at least in part, to be accessible to antibodies, making ribosomes antigenic particles which may function as decoys. Overall, our gPhage data provide a detailed map of the humoral response of patients with Chagas disease, validated by previous findings in the field (Alvarez *et al.*, 2001; Pitcovsky *et al.*, 2002; Motrán et al., 2000; Carmona et al., 2015), and expanding substantially the repertoire of antigens recognized in the context of this debilitating condition.

Our gPhage data may also shed light on a gap in knowledge regarding the antibody response during the course of Chagas disease. Anti-*T*. *cruzi* antibody levels in patients have been shown to correlate with parasite persistence and, eventually, disease resolution (Francolino et al., 2003; Bertocchi et al., 2013; Sabino et al., 2013). Treatment with the trypanocidal drug benznidazole (marketed as Rochagan or Radanil), one of the few therapeutic options for patients with chronic disease, leads to a decrease in anti-*T*. *cruzi* antibody levels two years after treatment (Álvarez et al., 2015). Antibodies to particular *T*. *cruzi* antigens have been proposed as correlates of therapeutic efficacy (Zrein et al., 2018; Buss *et al.*, 2020). Thus, the map of the antibody response that we have generated with gPhage may be a valuable tool for helping to improve and identify new biomarkers for Chagas disease. Although 4 of the selected epitopes identified in this study showed a notable correlation with the IgG pools from which they were identified (Figure 4B), none proved to be a specific marker for disease status (i.e., specific of patients who were asymptomatic or with CCC). However, at least 5 epitopes that we validated correspond to new epitopes not found in the IEDB database (cluster #7 Hypothetical protein PBJ77896; cluster #12 hypothetical protein EKG07867; cluster #15 protein phosphatase 2C RNF23118; cluster #19 hypothetical protein EKG03457; cluster #48 hypothetical protein ESS63713, and cluster #415 calpain-like cysteine peptidase PBJ79197), attesting to the validity of gPhage as a new platform for antigen/epitope discovery.

Finally, in the wake of the consecutive emerging/reemerging viral pandemics since the early 2000s, culminating in the present COVID-19 pandemic, we suggest that gPhage could be used to rapidly and efficiently map immunodominant epitopes in severe acute respiratory syndrome coronavirus 2. This could enable the timely deployment of diagnostic tools. Coupled to pathogen neutralization assays, the identification of immunodominant epitopes could accelerate vaccine development and production of therapeutic monoclonal antibodies for COVID-19, as well as in future emerging/reemerging and/or neglected infectious diseases. The limitations to the application of this technology are related to genome complexity: with larger introns and smaller exomes, it is possible that not all epitopes—especially those encoded by adjoining spliced exons—would be represented in the library. However, the technology could easily be expanded to eukaryotic pathogens whose genomes lack introns, such as *Leishmania* sp and others belonging to the family Trypanosomatidae. It could also be applied to pathogenic fungi with relatively simple genomes containing very few introns, such as those of *Candida* sp (Mitrovich et al., 2007). Indeed, with the appearance in the last decade of a multidrug resistant species of *Candida* (*C. auris*), gPhage could be an effective tool for studying the antibody response against this organism. The *Plasmodium falciparum* genome is similar in size to that of *T*. *cruzi* and only about half of the genes in *P*. *falciparum* contain introns, which are usually smaller than exons and less numerous than more complex life forms (Gardner et al., 2000). In this case, one could envision supplementing a *P*. *falciparum* gPhage library with custom synthetic DNA fragments to fill the gaps introduced by the presence of introns. In summary, we describe a high-throughput epitope identification tool that can be used to rapidly decipher the antibody response against emerging/reemerging and neglected infectious diseases.

### Limitations of the study

As in any novel enabling platform methodology and initial proof-of-concept, full validation of the putative epitopes and full antigens serially selected and isolated from our patient discovery cohort should ideally be experimentally confirmed in an independent patient validation cohort. Within this framework, the tentative antigens presented (Data S1) should be considered only as a candidate until unequivocally proven experimentally: They have merely been assigned to each individual epitope by using a software algorithm, which selected the highest-score antigen retrieved during database searches against all available *T*. *cruzi* genomes. As such, a few other potentially confounding factors merit further comment. First, while most of such epitopes likely represent a true corresponding antigen, the highest score antigen might belong to other related trypanosomatids (such as *T*. *cruzi* marinkellei antigens in bats, for instance) in a few cases. Moreover, in most cases, these same epitopes may also share high similarity to antigens belonging to other *T*. *cruzi* strains with more abundantly annotated genomes (yet another potential source of dry-lab bias). Indeed, in this report, each individual epitope was compared by pairwise alignment to curated member of these families to study the immune response against members of the multigene families. Finally, discrepancies in antigen assignment may conceivably reflect genomic differences among the various strains of *T*. *cruzi*.

Therefore, we would recommend that for follow-up studies, each individual epitope should be carefully curated with an appropriate software algorithm (and ultimately investigator supervision and discretion) for proper candidate antigen assignment. Of course, the actual clinical strains of parasites that infected the human patients used in this study are bound to be genetically different from the experimental standard strain used to build the gPhage library and those available in databases required for antigen assignment. Thus, our list of tentative epitopes and candidate antigens may reflect such biological diversity; in other words, a collection of epitope/antigen pairs that might share high similarity with the original immunogenic parasite protein in first place. Future studies with gPhage libraries build with clinical *T*. *cruzi* strains (perhaps even patient-specific or “personalized”) and comprehensive genome databases online and improvements in genomic annotation software should help mitigate these practical issues.

Given that this initial methodology and proof-of-concept report has also become a potential resource for other investigators, we have reasoned that we should include all candidate epitopes (including all singletons, which are epitopes defined by a single-peptide motif). Ongoing and future studies will certainly be required to assess whether these low-abundance single-peptide epitopes represent genuine antigens or not. Thus, we have opted instead of use an arbitrary experimental cutoff (i.e., abundance or number of peptides in the cluster) to allow independent researchers to study the full data set by using their own criteria. On this rationale, we have tabulated the data set into an easily online-searchable spreadsheet file with all available data generated in our study (Data S1).

Finally, we have identified at least one putative conformational epitope, which could not be validated by ELISA by using a synthetic peptide but which adopts a similar conformation in solution or in the context of the phage capsid protein. Thus, it is possible, perhaps even likely, that other selected epitopes from our list (Table 1), which were not validated by ELISA, may also correspond to conformational peptides and antigens that do not bind to the microtiter plate. In such setting, phage-ELISA might perhaps be an interesting complementary alternative to begin to study these epitopes. If so, researchers would have to isolate a targeted phage display particle for each specific epitope (or to clone them individually, if they are low abundance), which may practically limit the number of epitopes to be validated. As such, development of other high-throughput molecular or biochemical tools would be desirable.

## STAR★Methods

### Key resources table

**Table:** 

REAGENT or RESOURCE	SOURCE	IDENTIFIER
**Antibodies**		

Anti-Human IgG (γ-chain specific)−Peroxidase antibody produced in goat	Sigma-Aldrich	Cat#A6029; RRID AB_258272
IRDye® 680LT Goat anti-Rabbit IgG Secondary Antibody	Li-Cor	Cat#926-68021; RRID AB_2713919
Anti-fd Bacteriophage antibody produced in rabbit		Cat#B7786; RRID AB_258631

**Bacterial and virus strains**		

M13KO7 Helper Phage	New England Biolabs	Cat#N0315S
E. coli TG1	Lucigen	Cat#60502
E. coli DH10B	Invitrogen	Cat#18297010

**Biological samples**		

Sera from chagasic and healthy donors	University of Sao Paulo Medical School	N/A

**Chemicals, peptides, and recombinant proteins**		

Synthetic peptide for epitope validation in ELISA	Chinese Peptide Company	N/A

**Experimental models: organisms/strains**		

*Trypanosoma cruzi* Sylvio strain	Gift from Dr. Bianca Zingales (Chemistry Institute, University of Sao Paulo, Brazil) http://iq.usp.br/portaliqusp/?q=en	N/A

**Oligonucleotides**		

Illumina sequencing oligonucleotides (Sequences at Supplementary Data Table S4)	Exxtend (Brazil)	N/A

**Recombinant DNA**		

pG8SAET plasmid	Department of Microbiology, Swedish University of Agricultural Sciences, Uppsala	GenBank: AF130864.1
gPhage library	This paper	N/A
Software and algorithms		
BLAST	https://ftp.ncbi.nlm.nih.gov/blast/executables/blast+/	v 2.6.0
PEAR	https://cme.h-its.org/exelixis/web/software/pear/doc.html	v 0.9.10
MAFFT	https://mafft.cbrc.jp/alignment/software/	v 7.307
Jalview	https://www.jalview.org	v 2.11.0
MUSCLE	https://www.ebi.ac.uk/Tools/msa/muscle/	3.8.31
Chimera	https://www.cgl.ucsf.edu/chimera/	V 1.11
XSTREAM	https://amnewmanlab.stanford.edu/xstream/	v 1.73
FuzzyWuzzy	https://github.com/seatgeek/fuzzywuzzy	v 0.8
HMMER	http://hmmer.org/download.html	3.1b2
Sequence analysis script	This article	Data S3
Clustering script	This article	Data S3

### Resource availability

#### Lead contact

Further information and requests for resources and reagents should be directed to R.J.G (giordano@iq.usp.br).

#### Materials availability

Materials generated in this study are available upon request from the lead contact.

#### Data and code availability

The published article includes all data sets and code generated during this study (Data S3 and Table S2).

### Experimental model and subject details

#### Patients

The Institutional Review Board at the University of São Paulo Medical School approved the use of serum samples used in this study (approval number 0265/10). All patients with Chagas disease (N = 59) had at least two positive results for the presence of anti-*T*. *cruzi* antibodies. Patients with Chagas disease underwent electrocardiography and echocardiography and those with abnormal EKG (either right bundle branch block or left anterior fascicular block) were classified as having either mild cardiomyopathy, when LVEF was higher than 40% (LVEF> 40%), or severe cardiomyopathy, when LVEF was lower or equal 40% (LVEF ≤ 40%). Patients with no EKG alteration were considered asymptomatic. Serum samples were pooled into sets of 10 patients (2 groups, A and B, for each condition) and the immunoglobulins purified by affinity chromatography by using sepharose-G protein resin, following a standard manufacturer protocol (Thermo Fischer). All information regarding patients used in this study, including age and gender, can be found in Table S1.

#### Microbial strains

The epimastigote form of *T*. *cruzi* was grown in Liver Infusion Broth medium at 28°C (strain Sylvio X10, provided by Dr. Bianca Zingales) (Alves et al., 1986; Giordano et al., 1999). *E*. *coli* strain TG1 (Lucigen) and DH10B (Invitrogen) were used for phage display and cloning experiments, respectively.

#### Phagemid vector

The pG8SAET vector (GenBank: AF130864.1) was provided by Dr. Lars Frykberg (Department of Microbiology, Swedish University of Agricultural Sciences, Uppsala) (Zhang et al., 1999).

### Method details

#### *T*. *cruzi* culture and genomic DNA extraction

The epimastigote form of *T*. *cruzi* was grown in Liver Infusion Broth medium at 28°C (strain Sylvio X10) (Alves et al., 1986; Giordano et al., 1999). The cells were centrifuged and washed three times with phosphate-buffered saline (PBS), resuspended in lysis buffer containing 1% sodium dodecyl sulfate and 100 μg/ml proteinase K. The material was incubated for two hours at 50°C, centrifuged, and the supernatant recovered. Two extractions were performed with phenol:chloroform followed by precipitation of the DNA with ethanol. The DNA was re-suspended in TE buffer (10 mM Tris pH 8.0, 1 mM EDTA).

#### Genomic fragments generation

The purified genomic DNA was subjected to fragmentation in the COVARIS S2 equipment (Covaris Inc.). Aliquots of 30 μg in 130 μl of TE were fragmented at the following conditions: duty cycle, 10%; intensity, 4; cycles/burst, 200; time, 80 seconds. The fragments were subjected to electrophoresis in low-melting-point agarose gel and the region between 100 and 500 bp was selected and purified by extractions with phenol and chloroform. For DNA end repair, we treated the fragments with T4 DNA polymerase (Thermo Fisher Scientific) following a standard manufacturer protocol.

#### Production of cloning vector pG8SAET

Plasmid pG8SAET was propagated into *E*. *coli* strain DH10B (Invitrogen) and cultured in LB medium for 16 h. Plasmid DNA was isolated by using QIAGEN Plasmid Maxi Kit, following a standard manufacturer protocol. The material was subjected to two consecutive cesium chloride (CsCl)/ethidium bromide gradient purification, followed by extractions with n-butanol to remove ethidium bromide and dialysis against TE buffer for salt removal.

#### gPhage library production

The pure pG8SAET vector was digested with the enzyme Eco105I (Thermo Fisher Scientific) and dephosphorylated with FastAP (Thermo Fisher Scientific) following a standard manufacturer protocol. The vector was subsequently purified by phenol:chloroform extraction and ethanol precipitation to remove enzymes and other contaminants. The *T*. *cruzi* DNA fragments were ligated to the vector by using T4 DNA ligase (Thermo Scientific) following a standard manufacturer protocol of the enzyme. Several vector:insert molar ratios were tested to determine the best condition. In the final ligation reaction, we used 100 μg of the digested vector and a vector:insert ratio of 1:30. The DNA was transformed into electrocompetent *E*. *coli* bacteria (strain DH10B). The transformed bacteria were recovered and propagated overnight (ON) in LB medium containing carbenicillin (50 ug/mL). Serial dilutions were plated on LB agar medium to estimate the number of transformants in the library. In the next day, plasmid DNA from the library was purified, resuspended in TE and kept at -20°C until use. The plasmid library was transformed into *E*. *coli* (strain TG1 – Lucigen) and infected with the helper phage M13KO7 for phage production. All phage display selections were then performed using the same pool of gPhage library kept at -20°C (PBS solution, 50% glycerol) to minimize unwanted bias owing to phage amplification.

#### gPhage library coverage

The gPhage library contained ∼4x10^8^ unique inserts with average size of 143 bp. Sequencing data showed that ∼75% of the clones (∼3x10^8^) contained a *T*. *cruzi* insert with average size of 143 bp, which corresponds to a total of ∼4.3x10^10^ bp of the *T*. *cruzi* haploid genome (3x10^7^ bp) or ∼1,400 times. However, not all inserts result in peptide display and not all displayed peptides correspond to *T*. *cruzi* proteins (Figure 1F) (some peptides maybe derived from alternative reading frames of the genome). Taking into consideration that only 17.8% of the inserts encode a peptide and among those, only half (8.9%) share some degree of similarity (60%) a *T*. *cruzi* protein in databases, this still represents a coverage of approximately ∼100 times the encoded proteins within the parasite’s genome.

#### Phage display selection

Purified IgG (1 μg in 100 μl PB) was immobilized on microtiter plates (96 microwells) ON at 4°C, washed three times with PBS, blocked with PBS containing 1% bovine serum albumin (PBS/BSA) for 1 h at room temperature (RT), and incubated with phage library (for input, see Figure 2C) in 50 μl of PBS. To minimize the selection of phage display ubiquitous antigens, selection was performed in the presence of purified IgG (10 μg/ml) from the control group (added in solution together with the phage library). After 2 hours, wells were washed 10 times with PBS and phage bound to immobilized IgG recovered by bacterial infection (100 μl of *E*. *coli* TG1 strain in log phase). After dilution in LB media, a small aliquot was separated for quantification of phage recovery by colony count, and the remaining bacteria was then infected with helper phage at a multiplicity of infection of 20 and culture in LB media containing carbenicillin (100 μg/ml) (ON at 37°C, 300 rpm). On the following day, cells were centrifuged 10,000 *g* for 10 min, and phage particles were recovered from the supernatant by the NaCl/PEG method (Giordano et al., 2001). Three rounds of selection were performed after which, the pool of phage particles was used for large-scale sequencing. In all cases, the bacterial pellet was also recovered for plasmid purification.

#### Large-scale DNA sequencing

Sequencing of phage inserts was performed as described (Tang et al., 2019). In brief, plasmid DNA (10 ng) from the unselected library and from the third rounds of selection (control, asymptomatic, cardiomyopathy) was amplified by PCR with specific primers (Table S4). Four different forward and reverse primers were used (containing zero to three degenerated bases to add the diversity necessary for amplicon sequencing with the Illumina platform). The primers also contained an overhang corresponding to the sequence recognized by the Nextera XT kit. Phage were amplified for 20 cycles (melting: 95°C for 30 s; annealing: 55°C for 30 seconds; extension: 72°C for 1 min) with Kapa high-fidelity polymerase (Kapa Biosystems). All PCR products were purified (QIAGEN PCR purification kit) and a second PCR was performed to add the index adaptors (barcodes) by using the Nextera XT kit (Illumina) following a standard manufacturer protocol. The resulting libraries were quantified by qPCR by using the library quantification kit (Kapa Biosystems), diluted (4 nM), denatured (0.2M NaOH and 95°C for 5 min), and sequenced with the MiSeq Reagent Kit v2 (500 cycles) on an Illumina MiSeq equipment.

#### Bioinformatics

All scripts used in the study are available (Data S3). Paired-end reads were assembled with PEAR, and insert sequences were extracted, sorted as per their bar code sequences and counted (Zhang and Skolnick, 2004). Singletons were discarded and remaining sequences were aligned to all *T*. *cruzi* available genome sequences (strains CL Brener, Sylvio X10, DM28c, and Marinkellei) by using BlastN (Camacho et al., 2009) and those with less then 90% identity were discarded. Next, inserts that did not contain a full-length peptide (stop codons) were discarded, whereas remaining peptides were aligned to the protein database by using BlastP. Only peptides with at least 60% identity with *T*. *cruzi* proteins were used for further analysis (Data S3). For antigen clustering, identified *T*. *cruzi* peptides were first sorted by size (increasing order) and the first peptide (seed) removed from the list and compared with all remaining peptides (termed Levenshtein distance) by using the FuzzyWuzzy package (Levenshtein, 1966). Peptides with at least 80% sequence correspondence with the seed peptide are then removed from the list. The process continued until all peptides were clustered. Peptides with each cluster were aligned with Multiple Alignment using Fast Fourier Transform (Katoh and Standley, 2013) and the consensus sequence determined by using HMMER (Mistry et al., 2013) software package (http://hmmer.org/). Hydrophilicity analysis was performed by using available criteria (Parker et al., 1986) with a python algorithm. Repetitive sequences present in all *T*. *cruzi* available genome sequences (strains CL Brener, Sylvio X10, DM28c, and Marinkellei) were identified by using XSTREAM (Newman and Cooper, 2007). A HMM profile for each repetitive sequence was generated and compared by using HMMER to all identified antigens (Mistry et al., 2013). Alignments with expected values smaller than 10^-5^ were considered positive (Data S3).

#### Epitope mapping to multigene families

All 3,964 epitopes were compared by pair-wise alignment to representative members of the gp85/trans-sialidases, mucins, and MASP families. Only epitopes that were recognized by IgG from Chagas patients and shared at least 90% similarity were selected for the analyses. Members of the gp85/trans-sialidase sequences (N=417) (Freitas et al., 2011) were downloaded from TriTrypDB, aligned (MUSCLE, https://www.ebi.ac.uk/Tools/msa/muscle/) (Edgar, 2004), and sequences without the conserved FLY motive and Sialidase domain were not used. The MASP (N=1652) and mucin (N=468) sequences were downloaded from UniprotKB. MASP antigens without N- or C-terminal-conserved regions (De Pablos et al., 2016) and mucin sequences without the mucin conserved domains (Campo et al., 2004) were removed and not used for these analyses. To assist with domain identification, the epitopes were mapped to the corresponding sequences using Jalview (v2.11.0) (Waterhouse et al., 2009).

#### Ribosome 3-dimensional epitope mapping

All 3,964 epitopes were compared by pair-wise alignment to *T*. *cruzi* proteins (Genbank). Antigens that match *T*. *cruzi* ribosomal proteins were selected and aligned to proteins (blue) from the human ribosome (PDB #4UG0 and 4V6X—for the P2 protein). To locate their position in the structure, epitopes were visualized using Chimera (v1.11) (Pettersen et al., 2004).

#### ELISA

Synthetic peptides (Chinese Peptide Company) were immobilized on 96-well microtiter plate (50 μg/mL) in 50 mM carbonate buffer, pH 9.0 (OV at 4°C). Wells were washed with PBS supplemented with Tween-20 (0.05%) (PBST), blocked for 2 h at RT with PBS/BSA and incubated with patient sera in PBS (dilutions 1/100 up to 1/1,600). After 2 h at RT, wells were washed with PBST, incubated with goat anti-human IgG conjugated to horseradish peroxidase (Sigma Aldrich), washed with PBST and developed with OPD substrate (Sigma).

#### Phage-ELISA

IgG from individual patients (cohort A and B, severe CCC) were immobilized on 96-well microtiter plates (10 μg/ml, OV at 4°C). The wells were washed with PBS, blocked with PBS/BSA (1 h at RT) and incubated with 10^9^ transducing units of phage PPHTRRVTVRCGPPSCADE or negative control insertless phage (pG8SAET) in 100 μl PBS 1% BSA for 2 h at RT. For the competition assays, phage was incubated in the presence of synthetic peptide (10 μg/ml). Wells were washed and incubated with rabbit antibacteriophage IgG (Sigma, 1/400, 1 h at RT). Washed and incubated with goat anti-rabbit IgG conjugated to IRDye 680LT (LiCOR Biosciences) (1/1,000, 1 h at RT). Wells were washed and phage bound to the wells quantified using the Odyssey system (LiCOR).

### Quantification and statistical analysis

All statistical inference tests were performed by using GraphPad Prism 6. Tests used and corresponding *p*-values are reported along with each result as appropriate.

## Supplemental information

Document S1. Figure S1 and Tables S1–S4

Data S1. Excel file containing all epitopes, Related to Figure 3

Data S2. Alignments and consensus sequences for all epitopes. Related to Figure 3 and Data S1

Data S3. Python script to process large-scale DNA sequencing data (S1) and clustering peptides (S2), Related to Figures 1–3
