# Supplementary material for: A refined genome phage display methodology delineates the human antibody response in patients with Chagas disease
Source: iScience. 2021 May 15;24(6):102540. doi: 10.1016/j.isci.2021.102540 (PMC8185243; doi:10.1016/j.isci.2021.102540)
Supplement: Document S1. Figure S1 and Tables S1–S4 [file mmc1.pdf]

iScience, Volume *24*

Supplemental information

A refined genome phage display methodology delineates the human antibody response in patients with Chagas disease

André Azevedo Reis Teixeira, Luis Rodriguez Carnero, Andréia Kuramoto, Fenny Hui Fen Tang, Carlos Hernique Gomes, Natalia Bueno Pereira, Léa Campos de Oliveira, Regina Garrini, Jhonatas Sirino Monteiro, João Carlos Setubal, Ester Cerdeira Sabino, Renata Pasqualini, Walter Colli, Wadih Arap, Maria Júlia Manso Alves, Edécio Cunha-Neto, and Ricardo José Giordano

Table S1. Details for all patients used in this study (Chagas disease stage, gender, age and heart condition). Related to **Fig. 2** and STAR Methods (section: Patients).

Clinical Stage	Cohort	Number of individuals	Gender (male/ female)	Age (median, min, max)	Ejection fraction (median, min, max)
Asymptomatic	A	20	3M / 7F	57 (42 - 68)	0.67 (0.52 - 0.82)
	B	20	6F / 4M	60 (51 - 83)	0.65 (0.53 - 0.81)
Cardiac light	A	20	6M / 4F	53 (40 - 88)	0.68 (0.61 - 0.73)
	B	20	8F / 2M	49 (31 - 73)	0.70 (0.60 - 0.72)
Cardiac chronic	A	20*	6M / 4F	52 (34 - 88)	0.29 (0.18 - 0.35)
	B	20*	6F / 4M	48 (28 - 69)	0.30 (0.18 - 0.35)

* One female patient, 50 year of age with 0.18 ejection fraction was repeated in both cohorts.

Table S2. Sequencing details. Related to **Fig. 2**.

sample	# DNA reads [a]	# DNA reads with insert identi-fied [a]	# different DNA inserts [b]	# DNA reads with insert identified (no singleton) [a]	# different DNA inserts (no singleton) [c]	# DNA reads above genome identity cutoff (>=90%) [c]	# different DNA reads above genome identity cutoff (>=90%) [d]	# different DNA inserts encoding a peptide [d]	# different peptides encoded [e]	# peptide reads above proteome similarity cutoff (>=60%) [e]	# different peptide reads above proteome similarity cutoff (>=60%) [f]
Library	222,618 (100%)		222,099 (99%)			204,453 (92%)	204,021 (92%)				
control A	453,679 (100%)	420,109 (93%)	126,178 (30%)	330,712 (73%)	36,781 (11%)	321,890 (97%)	35,675 (11%)	153,266 (48%)	17,452 (11%)	41,640 (27%)	5,496 (31%)
control B	574,892 (100%)	541,438 (94%)	84,675 (16%)	474,143 (82%)	17,380 (4%)	471,831 (100%)	17,074 (4%)	62,358 (13%)	2,612 (4%)	10,856 (17%)	657 (25%)
asympto. A	455,717 (100%)	426,265 (94%)	143,331 (34%)	322,852 (71%)	39,918 (12%)	316,664 (98%)	38,884 (12%)	199,143 (63%)	20,495 (10%)	78,985 (40%)	8,182 (40%)
asympto. B	744,082 (100%)	704,653 (95%)	77,529 (11%)	645,124 (87%)	18,000 (3%)	460,752 (71%)	17,546 (4%)	353,541 (77%)	7,357 (2%)	200,056 (57%)	5,385 (73%)
mild A	868,305 (100%)	815,584 (94%)	214,287 (26%)	656,427 (76%)	55,130 (8%)	645,498 (98%)	53,574 (8%)	426,686 (66%)	28,244 (7%)	201,599 (47%)	11,472 (41%)
mild B	651,503 (100%)	609,289 (94%)	110,192 (18%)	523,434 (80%)	24,337 (5%)	519,622 (99%)	23,979 (5%)	248,547 (48%)	11,628 (5%)	232,266 (93%)	10,299 (89%)
severe A	1,134,445 (100%)	1,070,739 (94%)	194,714 (18%)	925,658 (82%)	49,633 (5%)	917,476 (99%)	48,464 (5%)	675,126 (74%)	24,341 (4%)	508,482 (75%)	10,425 (43%)
severe B	516,823 (100%)	497,444 (96%)	15,363 (3%)	486,343 (94%)	4,262 (1%)	484,296 (100%)	4,172 (1%)	480,049 (99%)	2,685 (1%)	427,369 (89%)	2,195 (82%)

a: relative to # DNA reads a

b: relative to # DNA reads with insert identified

c: relative to # DNA reads with insert identified (no singleton)

d: relative to # DNA reads above genome identity cutoff (>=90%)

e: relative to # different DNA inserts encoding a peptide

f: relative to # different peptides encoded

Table S3. Antigens with tandem repetitive sequences. The maximum number of repetition units in a single protein is indicated. Related to **Fig. 3 and 6**.

Repetitive unit	# repetitions	Antigen
PKPAE	177	EAN91735.1 trans-sialidase
EEAEARRLA	106	EAN83595.1 Tb-291 membrane-associated protein-like
EAEERAQR	103	EAN81788.1 membrane associated protein, putative
EDVQELVAPA	79	EAN89929.1 hypothetical protein, conserved
KQRAAEATKVAEAE	76	EAN93943.1 hypothetical protein
PFGQAAAGDKPS	72	EAN91665.1 surface antigen 2 (CA-2), putative
GSSMSEGSSHERSEG	65	EAN86927.1 hypothetical protein
PAEPKSAEPK	59	EAN87106.1 trans-sialidase
LAQREADNEKLAED	49	EAN86336.1 myosin heavy chain, putative
AAGGFGSATTTSAP	44	EAN88361.1 hypothetical protein
DSSAHSTPSTPV	40	EAN84377.1 trans-sialidase
ENERLADELEQKAA	40	EAN81003.1 hypothetical protein, conserved
ERQLQERDDALAALRDRLEEY<br>GREKSALESRSSESVDALAAL	38	EAN88119.1 hypothetical protein
LQRQNEELQSQLKESRRGEEKLDA	34	EAN99975.1 antigenic protein, putative
GFRG	31	EAN86592.1 nucleolar RNA-binding protein
AKPPAESPFKNVFGAPSSTA	28	EAN97077.1 hypothetical protein,
GFGDRGGR	24	EAN95753.1 hypothetical protein
TITTKVPTTTTTTTMAPT	23	EAN82230.1 hypothetical protein Tc00.1047053510433.10
ENEELRAEDEHKTRGLQ<br>EVSEQAEDLQRQLEELRA	21	EAN94898.1 hypothetical protein
LNDGGQRYSLSISVCVFTH<br>WTPNNPELSAAWRNFASSCPHG<br>CCTRLIVFERLLHHTLWSKFLFPI	21	EAN84488.1 hypothetical protein
QAARQQEDVQELVA<br>PAENVRELVAPTEDV	20	EAN98020.1 hypothetical protein, conserved
GPRHVDPDHFRSTTQDAYR<br>PVDPSAYKRALPQEEEEDV	19	EAN87716.1 microtubule-associated protein, putative
VTAEREELAENLRATEDAKAEVERNLES	17	EAN84693.1 kinesin-like protein
MEPTTITTKAPTTITT	17	EAN82230.1 hypothetical protein
GGQRGGGRRDYGDQGNQGDY	16	EAN87685.1 mitochondrial oligo_U binding protein TBRGG1, putative
MATDELTTEARPLDVTVGS<br>ALLSDSAAVTAPADRDATAL	14	EAN94669.1 hypothetical protein Tc00.1047053508119.200
TNDLQERAAAAEDAARRRC<br>AAAREKEEAAKRLEAELEVR	13	EAN84189.1 hypothetical protein
KPEEK	11	EKF26748.1 hypothetical protein
SAKKRPAVLSSQEVQAVVNA<br>RRATPRNDEYDDDYESETSA	10	EAN85405.1 hypothetical protein
GRGGDRGGFRG	10	EAN86596.1 nucleolar RNA-binding protein
KAAAPK	6	EAN99142.1 60S ribosomal protein L23a, putative
TTTTTTTTKAP	10	EAN83649.1 mucin TcMUCI
ASASEEGSRGDSSMP	9	EAN98211.1 trans-sialidase, putative
AAVPDSSSDDDDAPVR<br>KPAQKAKASPAMRPAPKK	8	EAN84654.1 hypothetical protein, conserved
STSSTPVGSGAK	8	EAN85648.1 trans-sialidase
NADDARSPLRRQPA	8	EAN85710.1 hypothetical protein, conserved

ARGQLVGEEREGTCGLHRDAV<br><br>DSEERAVRRCLERGEAAAVDELGEEYG<br><br>SATHERVVEALAAEEDA	8	EAN91803.1 hypothetical protein, conserved
KPEDAKPEEK	8	EAN83891.1 hypothetical protein
TKPVSSPP	8	EAN91857.1 hypothetical protein, conserved
LPKKKGRPKKLNEAEDAVAEM<br><br>TLMEEQEEMSAAVTEAEEAP	7	EAN91857.1 hypothetical protein, conserved
LTQEVQRLERELEEARREQERA<br><br>VGATEAMQRDAARERDEAVAA	7	EAN99422.1 hypothetical protein, conserved
LLEKDPRRNAKEIAALEESMNAR<br><br>AQELAREKKLADRAFLDQKPEGVSLR<br><br>ELPLDDDSDFVAMEQERRQ	6	EAN82258.1 calpain cysteine peptidase
RHSENADNVRSPLK	6	EAN85710.1 hypothetical protein, conserved

Table S4. Forward and reverse primers used for NGS sample preparation. Related to STAR Methods (section: Large-scale DNA sequencing)**.**

**FORWARD PRIMERS**	
ILLU-PG8-F-0N	TCGTCGGCAGCGTCAGATGTGTATAAGAGACAGCTGCGCAACACGATGAC
ILLU-PG8-F-1N	TCGTCGGCAGCGTCAGATGTGTATAAGAGACAGNCTGCGCAACACGATGACC
ILLU-PG8-F-2N	TCGTCGGCAGCGTCAGATGTGTATAAGAGACAGNNCTGCGCAACACGATGACC
ILLU-PG8-F-3N	TCGTCGGCAGCGTCAGATGTGTATAAGAGACAGNNNCTGCGCAACACGATGACC
**REVERSE PRIMERS**	
ILLU-PG8-R-0N	GTCTCGTGGGCTCGGAGATGTGTATAAGAGACAGGTTCCAGTGGGTCCGGATA
ILLU-PG8-R-1N	GTCTCGTGGGCTCGGAGATGTGTATAAGAGACAGNGTTCCAGTGGGTCCGGATA
ILLU-PG8-R-2N	GTCTCGTGGGCTCGGAGATGTGTATAAGAGACAGNNGTTCCAGTGGGTCCGGATA
ILLU-PG8-R-3N	GTCTCGTGGGCTCGGAGATGTGTATAAGAGACAGNNNGTTCCAGTGGGTCCGGATA

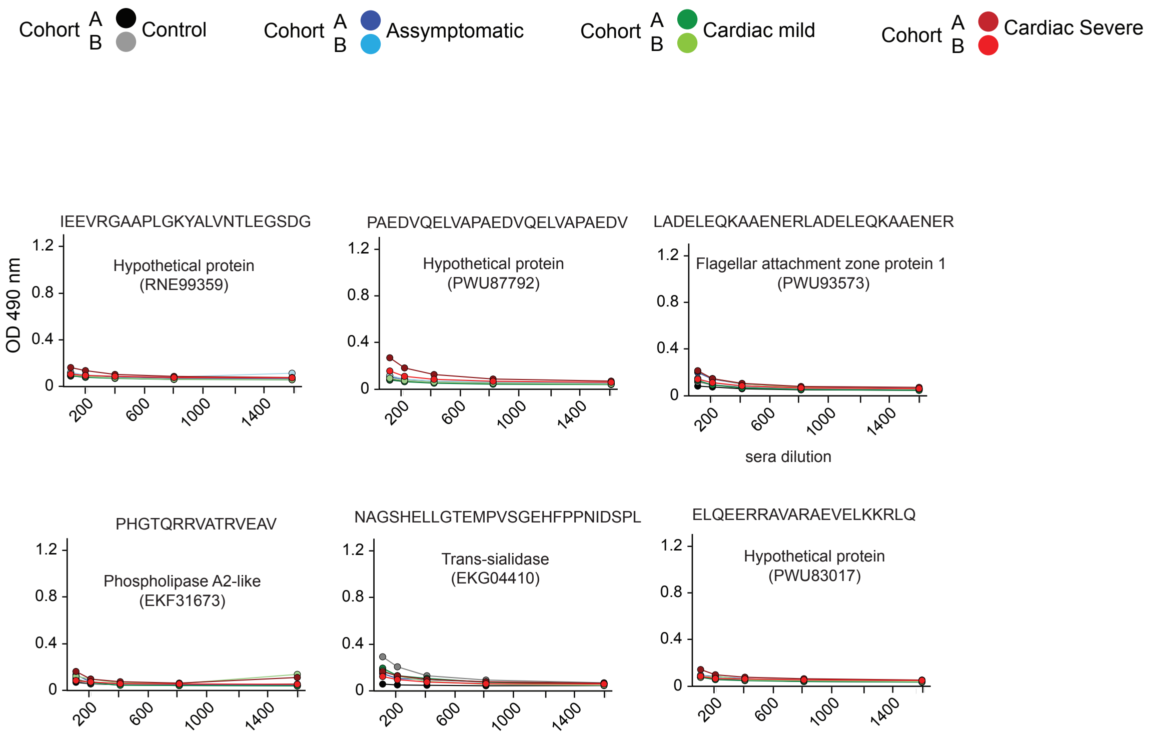

Figure S1. Antigen validation. ELISA reactivity of each pool of IgG from both cohorts with synthetic peptides representative of selected clusters of antigens. Related to **Fig. 4**.
